# Improving the p-Type CuCrO_2_ Thin Film’s Electrical and Optical Properties

**DOI:** 10.3390/ma16031000

**Published:** 2023-01-21

**Authors:** Jiaxin Jiang, Yu-Feng You, Dhanapal Vasu, Sheng-Chi Chen, Te-Wei Chiu, Gopi Prashanth, Po Chou Chen

**Affiliations:** 1 Department of Materials and Mineral Resources Engineering, National Taipei University of Technology, 1, Sec. 3, Zhongxiao E. Rd., Taipei 106, Taiwan; 2 Institute of Materials Science and Engineering, National Taipei University of Technology, No. 1, Sec. 3, Chung-Hsiao East Road, Taipei 106, Taiwan; 3 Department of Materials Engineering and Center for Plasma and Thin Film Technologies, Ming Chi University of Technology, New Taipei City 243, Taiwan; 4 Godi Energy, 12 (p), 13, 14 (p), Road No. 2, Hardware Park, Hyderabad 500005, India; 5 Graduate Institute of Organic and Polymeric Materials, National Taipei University of Technology, Taipei 106, Taiwan; 6 E-Current Co., Ltd., 10F.-5, No. 50, Sec. 4, Nanjing E. Rd., Songshan Dist., Taipei City 10553, Taiwan

**Keywords:** delafossite material, CuCrO_2_, thin film growth, energy efficiency, spin coating, microstructure

## Abstract

In this research, we studied the functional properties of CuCrO_2_, which is the most promising p-type transparent conductive oxide (TCO). The thin films were fabricated using a spin coating technique. The diffraction patterns were obtained with the help of X-ray diffractions, and the optical properties of absorption characteristics were studied using UV-visible absorption. The physical properties of film formation and surface morphology were analyzed using FESEM analysis. The aging properties were also analyzed with the help of various precursors with different aging times. The CuCrO_2_ thin films’ functional properties were determined by using chelating agent and precursor solution aging times. The CuCrO_2_ thin films have better transmittance, resistance, figure of merit (FOM), and electrical conductivity. Moreover, the resistivity values of the CuCrO_2_ thin films are 7.01, 9.90, 12.54, 4.10, 2.42, and 0.35 Ω cm. The current research article covers the preparation of copper chromium delafossite thin films. These thin films can be suitable for hole transport layers in transparent optoelectronic devices.

## 1. Introduction

Transparent conductive oxide (TCO) is a type of semiconductor material combining excellent electrical conductivity and optical transmittance towards the visible range. TCO-based thin films have been utilized in a wide variety of applications, such as touch screens, flat panel displays, and solar cells [1]. Delafossite materials have a high absorption coefficient, prolonged carrier lifetime, excellent charge mobility, and greatly increased power conversion efficiency (PCE) in the short term. Delafossite materials are ternary oxide compounds with the general formula of A_I_B_III_O_2_, where A belongs to monovalent cations, such as Cu or Ag, and B belongs to trivalent metals ranging from Al to La. CuCrO_2_ materials belong to the family of delafossite; they have excellent conductivity among the other delafossite materials. CuCrO_2_ materials are more stable and highly efficient compared to other alkali metal delafossite materials due to their physiochemical properties and electronic configuration of Cu^+^, Cr^3+^, and O^2−^ [1,2].

Perovskite solar cells (PSCs) are usually fabricated in a p-i-n or n-i-p structure, in which the perovskite layer of the n-i-p structure is sandwiched between the hole transport layer (HTL) and the electron transport layer (ETL). In addition, the top layer of PSCs is the hole transport layer [3]. The HTL layer plays a key role in PSCs to restrict the electron transport and extract holes. Moreover, when the energy levels of the perovskite layer and the hole transport layer are mismatched, the efficiency will change [4]. However, hydrophobicity and chemical stability will affect the HTL layer performance. Hence, these issues should be rectified to enhance the HTL’s performance [5]. Commonly, the HTLs are mostly made up of organic materials, such as Spiro-MeOTAD, PTTA, and P3HT, but these materials usually require additives to improve conductivity, resulting in damage to PSC devices and insufficient hole mobility. In addition, there is a high cost of raw materials and difficulty in the synthesis of these materials [6]. Moreover, inorganic materials have excellent stability whereas cost-effective.

The Cu-based delafossite belongs to p-type TCO, such as CuAlO_2_, CuCrO_2_, and CuFeO_2_, with a wide energy gap, high conductivity, high hole mobility, good optical transparency, and chemical stability [7]. The CuCrO_2_ materials’ transport mechanism is a small polaron of Cu^+^ and Cu^2+^ ions. Moreover, research studies have reported that the p-type conductivity is associated with the generation of defects, such as vacancy of Cu (VCu), which have lower generation energy. The Cu anti-site defects are due to the possible formations of Cu atoms occupying Cr sites. Indeed, the intrinsic-type dopants are proven to be responsible for the improvement of the charge carrier density, resulting in a change in electrical properties. Therefore, CuCrO_2_ has emerged as a viable HTL material due to its low-toxicity components and energy gap configuration which is compatible with the perovskite layer [8]. CuCrO_2_ has various advantages, such as low activation energy and high mobility [9,10]. The literature shows that the resistivity of delafossite is usually much lower in the ab plane than in the c-axis direction (PdCoO_2_, room temperature, ρ_c_/ρ_ab_ ∼ 411 [10]; CuCrO_2_, room temperature, ρ_c_/ρ_ab_ ∼35 [11]), while mobility also varies (μ_ab_ = 34 and μ_c_ = 8.9 cm^2^ V^−1^ s^−1^ [9]).

The preparation of TCO thin films influences their properties, such as grain size, shape, surface morphology, growth mode, and bandgap. Therefore, the synthesis techniques, the conditions, and the doping methods are important factors to be considered. Various preparation methods have been utilized to prepare the HTL of TCO thin films’ such as the spray pyrolysis method [12], the sputtering method [13], the spin coating method [14], the atomic layer deposition method [15], and other techniques. Among them, the spin coating method has great flexibility, is a simple process, has low cost, and is commercially viable [16]. Using the spin coating method, it is possible to attain a film with uniform growth, grain shape, size, and surface morphology. Moreover, the thin film growth parameters depend on the concentration of the precursor solution, the stirring and aging time of the sol–gel process, the preheating temperature, and the annealing method [17]. Hence, chemically stable, and non-toxic TCOs HTL (CuCrO_2_) can be prepared by using simple and low-cost methods, such as the sol–gel process.

Generally, the spin coating method uses different types of precursors, such as metal precursors (nanoparticles and ions), dispersants (volatile organic solvents, such as alcohol and acetone), and chelating agents [18]. The purpose of adding a chelating agent is to avoid or resist the hydrolysis reaction of the metal precursor in the dispersant, resulting in stability or uniform precipitation [19], which can increase the solubility of the metal salt and improve the uniformity of the precursor [20]. The chelating agent we chose for this experiment is 2-Aminoethanol (ETA). ETA forms a chelate complex with metal ions through -NH_2_ [21], which protects the metal ions in the precursor and it can control the size and agglomeration phenomenon [22].

To avoid the influence of substrate alignment on thin film epitaxy [23], high-penetration amorphous alkali-free glass was selected as the substrate in this experiment, and a hydrogen–nitrogen mixture gas was used for the two-step annealing, resulting in Cu^2+^ being reduced to Cu^+^ by H_2_ so that CuCrO_2_ can be formed at a lower-temperature transmittance of the film [24], and the aging time was controlled by a chelating agent to obtain the desired film grain and surface morphology.

In this article, we prepared the TCO HTL layer using CuCrO_2_ on the alkali-free glass substrate using the spin coating technique. The aging time was also studied by adding a chelating agent. The prepared materials were annealed under different atmospheres, such as hydrogen, nitrogen, and the hydrogen–nitrogen mixture atmosphere, respectively. The prepared thin film’s physio-chemical properties were studied by using various analytical techniques.

## 2. Materials and Methods

This study used copper nitrate [(Cu(NO_3_)_2_·3H_2_O), 99.0%, SHOWA], chromium nitrate, chromium acetate hydroxide [(CH_3_CO_2_)_7_ Cr_3_(OH)_2_, 99.0% ALDRICH], and 2-Methoxyethanol [HOCH_2_CH_2_OCH_3_, 99.0%, TEDIA]. All the chemicals were bought from Sigma Aldrich, Taipei, Taiwan.

### 2.1. Preparation of CuCrO_2_ Solution

The CuCrO_2_ film’s stability was observed by using a chelating agent with different aging times. Solution A was prepared as follows: copper nitrate and chromium nitrate were dissolved in 2-Methoxyethanol (10 mL) at a molar ratio of 1:1 and stirred for 24 h to obtain a homogenous solution without a chelating agent. Sample B was prepared by using the above-mentioned process. In addition, the chelating agent was added to the solution and the solution was stirred for 24 h. Similarly, C, D, E, and F samples were prepared using the above-mentioned process while varying the aging time. This is shown in Table 1.

### 2.2. Preparation of CuCrO_2_ Thin Films by Spin Coating

**Figure 1:**
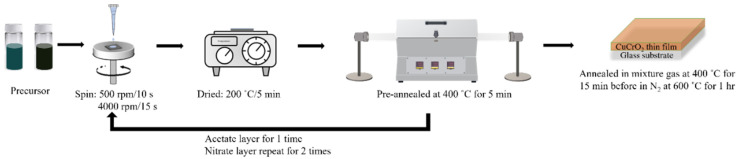
Schematic diagram of the preparation of CuCrO_2_ film coating on the alkali-free glass substrate.

A glass plate was cut into small sizes (1.5 × 1.5 cm) and washed with ethanol and water for several times. Then, the thin films were coated on the substrate in three different layers. The first layer was coated using the copper nitrate solution with different rpm (500 and 4000) for 10 and 15 s, respectively. The second layer was coated by using the chromium nitrate solution, and the third layer was coated by using the above-prepared complex solution. After spin coating, the spin-coated film was heated on a hot plate at 200 °C for 5 min and dried. The sample was then calcinated at 400 °C for 5 min using a tubular furnace to eliminate the precursor residuals. Finally, the prepared thin films were annealed at 400 °C for 15 min under the atmosphere of mixed gases (H_2_/N_2_ 10:90 sccm) to reduce Cu^2+^ to Cu^+^. Thereafter, the annealed film was again annealed at 600 °C for 1 h in the N_2_ atmosphere to obtain the CuCrO_2_ thin films. The preparation methods are depicted in Figure 1.

### 2.3. Characterization of CuCrO_2_ Thin Films by Spin Coating

The CuCrO_2_ thin films’ characteristics were characterized by using various analytical characterization techniques. The CuCrO_2_ thin films’ crystalline structure was analyzed by using grazing incidence X-ray diffraction (GIXRD D8, Bruker ADVANCE, Billerica, MA, USA), and the range of 2-Theta was from 20° to 80° (the incident angle was 0.5°, Cu Kα λ = 1.5418 Å). The cross-sectional and surface morphology of the film was observed with the help of a field-emission scanning electron microscope (FESEM, FE-SEM/EDX, JEOL, JSM-7610F, and Hitachi Regulus 8100, Tokyo, Japan). The optical properties of the prepared films were measured using an ultraviolet-visible spectrometer (UV-Vis, Shimadzu 2600, Kyoto, Japan) at a wavelength range of 300–800 nm. The vibrational bonds and the functional groups of the prepared materials were studied by using a micro-Raman spectrometer (Micro-Raman Spectrum, ACRON, UniNanoTech Co., Ltd., Yongin, Republic of Korea) and a Fourier-transform infrared spectrometer (FTIR, Spotlight 200i Sp^2^ with Auto ATR System, Perkin Elmer, Inc. Waltham, MA, USA). The resistivity was measured by using a four-point probe (Four-Point Probe, Keithley 2400 SourceMeter^®^ SMU Instruments, Cleveland, OH, USA), and the carrier concentration was measured by using the hall method (Ecopia HMS-3000 Hall Measurement System, Gyeonggi-do, Republic of Korea).

## 3. Results

### 3.1. XRD Analysis

**Figure 2:**
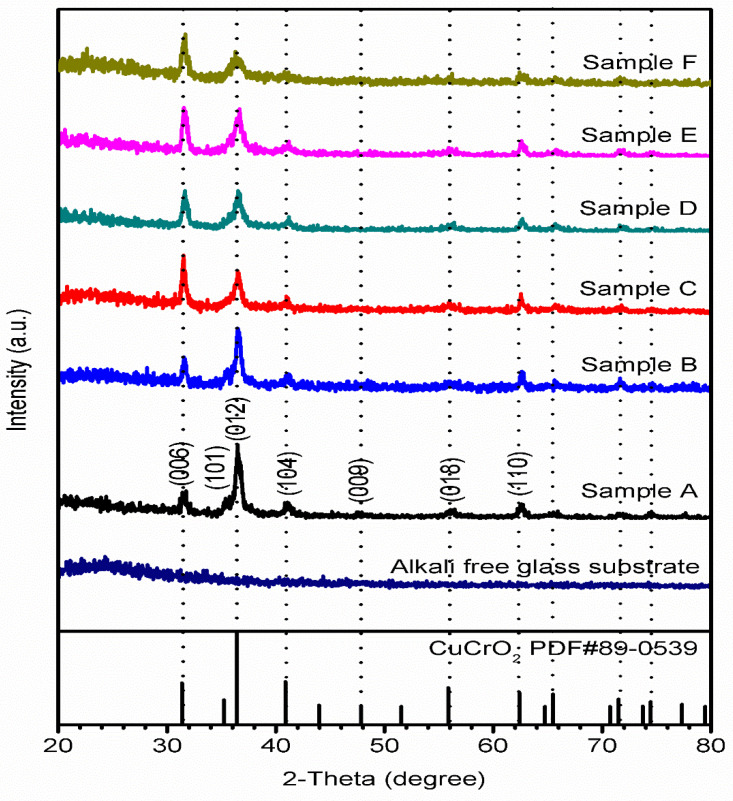
XRD patterns of alkali-free glass substrate, Sample A precursor without chelating agent added, Sample B precursor with 0 min aging time, Sample C precursor with 30 min aging time, Sample D precursor with 1 h aging time, Sample E precursor with 2 h aging time, and Sample F precursor with 4 h aging time which was prepared for CuCrO_2_ spin coating.

The GIXRD was used to study the CuCrO_2_ thin films’ crystal structure and diffraction patterns. Figure 2 shows the X-ray diffraction patterns of the CuCrO_2_ thin films. In the XRD analysis, we can observe various peaks at 31.36°, 35.21°, 36.41°, 40.87°, 47.83°, 55.85°, and 62.41° corresponding to (006), (101), (012), (104), (009), (018), and (110), respectively. The obtained crystalline planes are well matched with the JCPDS no PDF#89-0539 CuCrO_2_. Moreover, the crystalline peaks show that CuCrO_2_ is the main phase, and there are no impurity phases such as Cr_2_O_3_ and CuO. In addition, without 2-Aminoethanol, the peak at 36.41 (012) intensity is higher and the peak is very sharp. After adding the chelating agent and due to aging time, the diffraction planes (006) and (110) intensity decrease and the FWHM also increases. Similarly, all the diffraction plane’s intensity decreases and the FWHM increases in the samples D, E, and F. Therefore, the XRD diffraction patterns indicate that the CuCrO_2_ has been successfully formed. Using the Scherrer Equation, the crystallite size of the prepared thin films is calculated [1]. The formula is as shown below, where ε is the crystallite size; K is the Scherrer constant; λ is the wavelength of the incident radiation; b is the full width at half maximum (FWHM) of the crystalline peaks; and θ is the Bragg angle [1,2,3].

ε = Kλ/(bcos(θ))(1)

The prepared thin films’ average crystalline size is 15.2, 18.1, 17.9, 13.1, 12.8, and 10.3 nm, respectively.

### 3.2. SEM Studies

**Figure 3:**
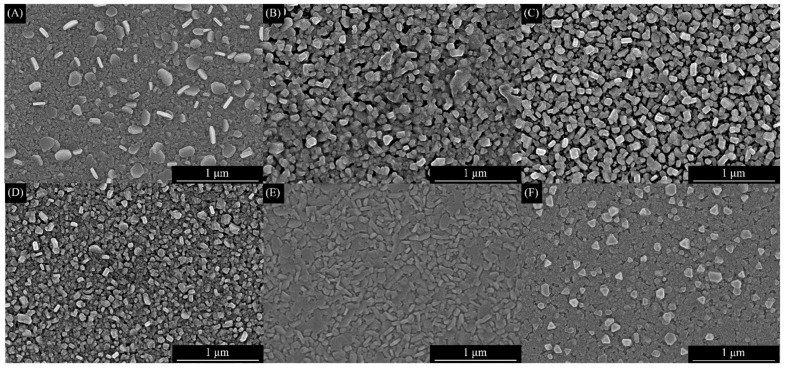
FESEM images of the top of the CuCrO_2_ thin films coated on the alkali-free glass substrate: (**A**) Sample A precursor without a chelating agent added, (**B**) Sample B precursor with 0 min aging time, (**C**) Sample C precursor with 30 min aging time, (**D**) Sample D precursor with 1 h aging time, (**E**) Sample E precursor with 2 h aging time, and (**F**) Sample F precursor with 4 h aging time which was prepared for CuCrO_2_ spin coating.

The prepared CuCrO_2_ thin films’ surface structure and morphology were observed using FESEM analysis. The surface morphologies of the annealed CuCrO_2_ thin films for different aging times are exhibited in Figure 3. The effect or influence of the chelating agent and aging time can be observed on the growth of grains. Figure 3 shows that the CuCrO_2_ without a chelating agent shows irregular grain shape and size. However, when a chelating agent is added to the precursor solution with different aging times, such as 0, 30 min, 1 h, and 4 h, it shows the irregular shape and size of the thin films. The precursor solution with an aging time of 2 h exhibits a homogenous surface morphology, uniform grain shape, and good crystalline size. This exhibits a similar changing trend to the results of XRD patterns. The prepared CuCrO_2_ films’ crystalline sizes are better and form hexagonal-shaped crystallites with uniform grain boundaries, which can be utilized to observed the prepared delafossite surface morphology.

**Figure 4:**
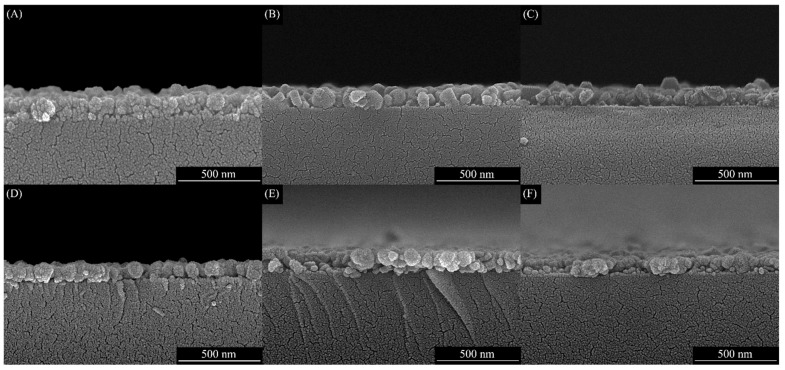
FESEM images of the cross section of the CuCrO_2_ thin films coated on the alkali-free glass substrate: (**A**) Sample A precursor without a chelating agent added, (**B**) Sample B precursor with 0 min aging time, (**C**) Sample C precursor with 30 min aging time, (**D**) Sample D precursor with 1 h aging time, (**E**) Sample E precursor with 2 h aging time, and (**F**) Sample F precursor with 4 h aging time which was prepared for CuCrO_2_ spin coating.

In Figure 4, the FE-SEM cross-sectional image of the CuCrO_2_ thin films and the thickness of the prepared films can be observed. Noticeably, the spin-coated film is tightly packed to the substrate with a uniform thickness of 142 nm (Table 2). Though the chelating agent was added at different aging times, all films portray almost similar cross-sectional surface morphology due to the same preparation methods. However, the thin film thickness varies with the precursor solution aging time, and the CuCrO_2_ thin film thicknesses are 120, 122, 111, 139, and 112 when the precursor solution aging times are 0 min, 30 min, 1 h, 2 h, and 4 h, respectively.

### 3.3. UV-Vis Analysis

**Figure 5:**
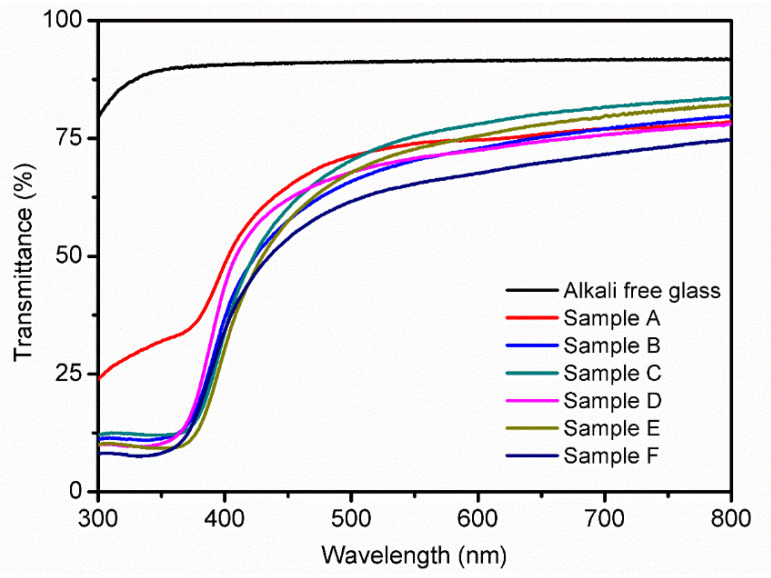
UV-Vis spectrum of the CuCrO_2_ thin films coated on the alkali-free glass substrate with different aging-time precursors.

The visible-light transmittance is the most important parameter for the applications of TCO thin films. The prepared CuCrO_2_ thin films’ optical transmittance was investigated using an UV-Visible spectrometer in the wavelength range of 300–800 nm (Figure 5). The CuCrO_2_ thin films’ transmittance changes in the visible region and varies with varying precursor solution aging time owing to the enhancement in the crystallinity and grain size. The lower-transmittance films may be the result of photon scattering by the film grain boundaries or pores owing to poor crystallization. The prepared thin films’ transmittance values are presented in Table 3. As a result of UV-Vis, the high transmittance in the visible-light region shows that the prepared CuCrO_2_ thin films have a better potential for applications in perovskite solar cells.

The absorption coefficient (α) can be calculated from the transmittance data of the prepared samples as follows [1,2,3]:

α = (1/d)ln(1/T)(2)

where d is the thickness of the film and T is the transmittance of the film. According to the following equation, the absorption coefficient (α) and the photon energy (hν) of the material can be calculated as follows [25]:

αhν = A(hν − Eg)^1/2^ (3)

**Figure 6:**
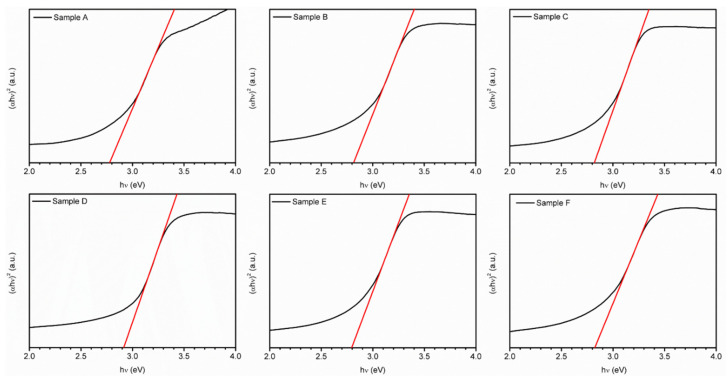
The Tauc plot of the CuCrO_2_ thin films coated on an alkali-free glass substrate: Sample A’s direct band gap is 2.78 eV, Sample B’s direct band gap is 2.81 eV, Sample C’s direct band gap is 2.82 eV, Sample D’s direct band gap is 2.91 eV, Sample E’s direct band gap is 2.79 eV, and Sample F’s direct band gap is 2.82 eV.

Figure 6 shows the energy band gap values calculated from the Tauc plot for the transmission spectrum of each sample. It can be observed that the absorption peaks of the prepared films are all in the range close to visible light, showing that they have good transmittance in the visible light range. Among them, sample C with an aging time of 30 min has the highest transmittance of 73.8%, and a direct energy gap from 2.78 to 2.91 eV, which is consistent with the literature [26,27]. The curve of Sample A, which is between 300 and 370 nm, can be considered as the non-uniform distribution of metal ions due to the absence of a chelating agent, resulting in relatively poor and certain impurities in the film prepared by coating.

### 3.4. FT-IR Analysis

**Figure 7:**
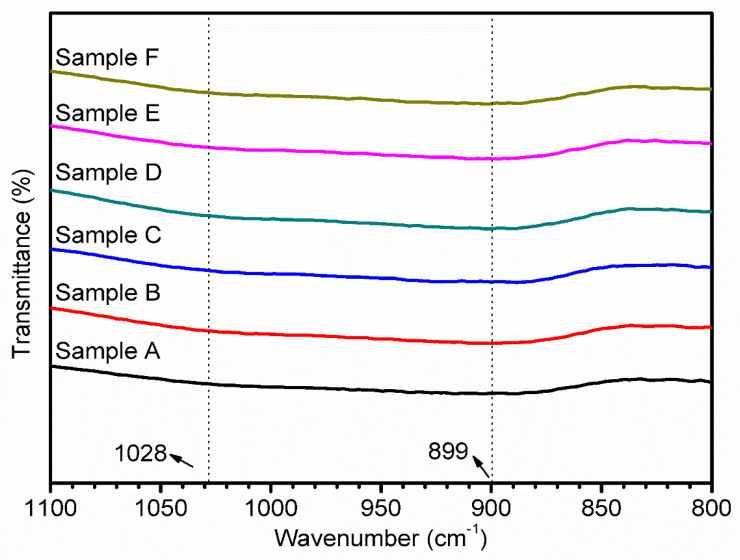
FT-IR spectrum for the CuCrO_2_ thin films coated on the alkali-free glass substrate.

The FT-IR spectrum of the CuCrO_2_ thin films is shown in Figure 7. There are bending vibrations of the Al-O bond in the [AlO_6_] octahedron at 580 cm^−1^ [28], and 778 cm^−1^ [29] is the bond of [BO_3_]. The bending vibration of the B-O-B bond in the triangular unit [30] and the stretching vibration of Cr-O are observed at 778 cm^−1^, which corresponds to the stretching vibration of Cu-O [31]. The bending vibration of the Al-O-Al bond in the [AlO_4_] tetrahedral unit and the symmetrical stretching vibration of the Si-O-Al bond appear at about 899 cm^−1^ [28,29], and Cr-stretching vibrations of O-M bonds are also observed [31]. The peaks 938 cm^−1^ can be attributed to the stretching vibrations of Si-O bonds and the symmetrical stretching vibrations of Si-O-Si bonds [32]. The signal at about 1028 cm^−1^ corresponds to the asymmetric stretching vibration of the Si-O-Si bond in the [SiO_4_] tetrahedral unit or the stretching vibration of the B-O bond in the [BO_4_] tetrahedral unit [32]. The 1374 cm^−1^ signal corresponds to the asymmetric stretching vibration of the B-O bond in the [BO_3_] triangular unit [30]. It can be seen from this that the relative thickness of the CuCrO_2_ films is only about 110–150 nm, and the signal of the alkali-free glass substrate itself [33] is extremely strong. However, we have observed the solvent of 2-Methoxyethanol related peaks at 3400–3500 cm^−1^ and 2700–3000 cm^−1^. Moreover, after annealing we cannot observe these materials related peaks.

### 3.5. Raman Studies

The delafossite material CuCrO_2_ and other delafossite materials commonly have four atoms in the unit cell, which will produce 12 vibrational Raman modes:

Γ= A_1g_ + E_g_ + 3A_2u_ + 3E_u_ (4)

**Figure 8:**
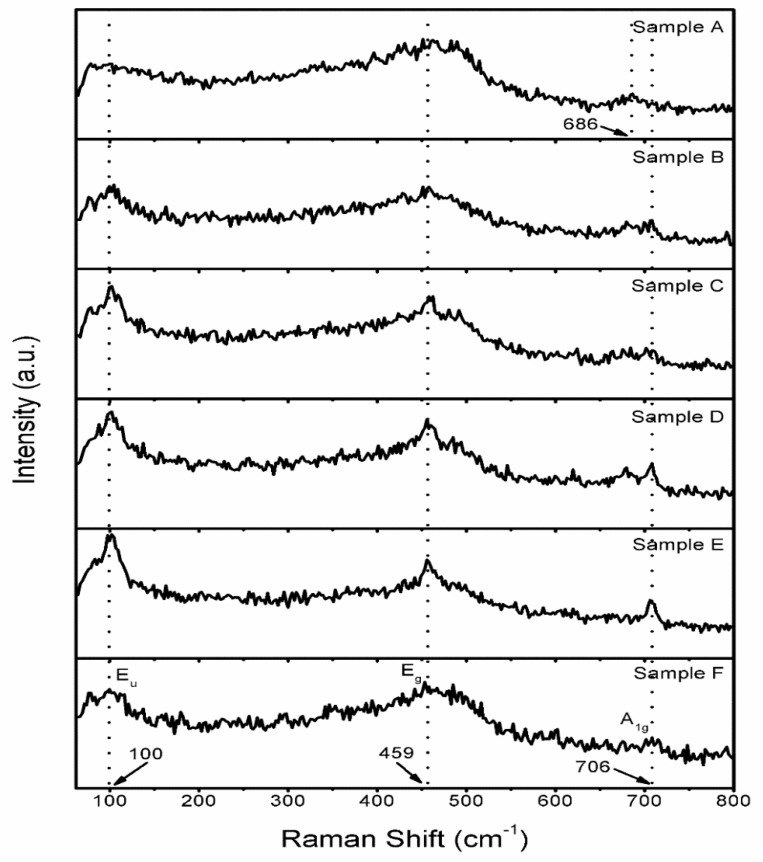
Raman spectra of the CuCrO_2_ thin films coated on the alkali-free glass substrate under the excitation line of 532 nm.

Three of them are acoustic modes, nine are optical modes, and A_1g_ and E_g_ are the symmetrical Raman-active modes [34]. The A mode is the vibration along the c-axis, which is along the direction of the Cu-O bond, and the E mode represents the vibration in the triangular lattice perpendicular to the c-axis [35] The normal modes are categorized according to their parity when an inversion center exists in the structure. The subscript u stands for odd modules, such as A_2u_ and E_u_. The literature shows that when Γ is in an odd mode, two oxygen atoms will move in the same direction, while Cu and Cr atoms will not be affected [36,37]. According to Figure 8, the Raman scattering peaks of the CuCrO_2_ films are around 100 cm^−1^ (E_u_), 459 cm^−1^ (E_g_), and 706 cm^−1^ (A_1g_), which is consistent with the literature; however, at about 212 cm^−1^, the A_g_ peak recorded in literature does not appear [38]. It can be seen from Figure 8 that Sample A without a chelating agent has an obvious frequency shift to a lower wave number at the position of A_1g_, which is due to the weakening of the Cr-O bond [38]. Samples B-F after adding the chelating agent all have the E_u_ peaks, but the peak of Sample F is wider, which may be the same as that of Sample A, because of the smaller grain size and lower crystallinity [39]. According to the literature, the stronger the A_g_ peak, the more grains the sample grows along the parallel c-axis [40]. Therefore, Figure 8 can prove that after adding the chelating agent, the crystal grains on the surface of the film grow parallel to the c-axis, and most of them grow along the direction perpendicular to the c-axis. From Samples B-E, with the aging time of 2 h, the crystallinity of the film is the highest and most of the film grows in the direction perpendicular to the c-axis, which is consistent with the SEM image.

## 4. Electrical Properties and Figure of Merit (FOM)

The prepared CuCrO_2_ thin films’ electrical properties were studied for all samples, which are tabulated in Table 4. The resistivity values vary with the chelating agent and aging time. These results suggest that the aging time and thickness variation affect the electrical conductivities. The lowest resistivity value is obtained in the Sample F and the resistivity value is 0.3 Ω cm. In addition, the FOM values are also calculated and tabulated in Table 4. To understand the effect of thickness on the electrical properties, the resistivity was measured by using a four-point probe (Four-Point Probe, Keithley 2400 SourceMeter^®^ SMU Instruments), and the carrier concentration was measured by using the hall method (Ecopia HMS-3000 Hall Measurement System). The figure of merit (FOM) as proposed by Haacke was used to calculate the film quality, where T is the average transmittance in the visible light range, and R_sh is the sheet resistance of the film [5]:

φ = T^10^/R_sh (5)

The results show that the samples all exhibit p-type conductivity. Their electrical properties are shown in Table 2. It can be seen that Sample A has higher conductivity, but the carrier concentration is lower, while Samples B and C have higher carrier concentrations, but the sheet resistance is also higher. This may be because the surface of the film is discontinuous due to the growth of lamellar crystals on the film surface. In Samples D–F, as the aging time increases, the sheet resistance of the film decreases from 4.10 Ω cm to 0.35 Ω cm, and the FOM also increases from 8.21 × 10^−6^ Ω to 4.32 × 10^−5^ Ω. This may be because the film surface is more continuous. However, Sample E has a higher carrier concentration. Finally, Table 5 compares the photoelectrical properties of Sample E and Sample F in this experiment with other CuCrO_2_ films reported in the literature. The films used in this study have good light transmittance, good conductivity, and excellent electrical properties.

## 5. Conclusions

The delafossite CuCrO_2_ thin films were successfully prepared by a simple spin coating method. The basic characterizations were successfully utilized to study the electrocatalytic aspects of copper delafossite CuCrO_2_ thin films. The reaction kinetics and mechanism of these prepared materials in the solid-state reaction were observed and it is strongly depending on the presence of Cu^+^ ions with respect to the annealing process. Samples E and F exhibited excellent physio-chemical properties compared to the other delafossite materials. Indeed, the obtained results are better than already reported literatures. In addition, the proposed process obtained a higher p-type FOM value of 4.32 × 10^−5^. The improvement of p-type conductivity is attributed to the generation of the intrinsic defects with Cu, created Cu anti-site defects have occupy the Cr vacancies, which leads to the film crystallinity. In the current research work, it resulted in the enhancement of the optical and electrical properties, such as mobility, resistivity, transmittance, crystallinity, and FOM. This process allows us to prepare potential candidates for TCO’s HTL layers, organic devices, organic light-emitting diodes (OLEDs), and perovskite solar cells.

## Figures and Tables

**Table 1:** The spin coating conditions for CuCrO_2_ thin film preparation.

Sample	Precursor Aging Time
A	No chelating agent added
B	0 min
C	30 min
D	1 h
E	2 h
F	4 h

**Table 2:** The average thickness of the CuCrO_2_ thin films.

Sample	Thin Film Thickness
A	142 nm
B	120 nm
C	122 nm
D	111 nm
E	139 nm
F	112 nm

**Table 3:** The average transmittance of the CuCrO_2_ thin films in the visible range.

Sample	Transmittance (%)	Band Gap (eV)
A	72.6	2.78
B	69.8	2.81
C	73.8	2.82
D	70.5	2.91
E	71.4	2.79
F	65.0	2.82

**Table 4:** The electrical properties and FOM of the CuCrO_2_ thin films.

Sample	Thickness (nm)	Sheet Resistance (Ω/cm^2^)	Resistivity (Ω cm)	Carrier Concentration (cm^−3^)	FOM (Ω^−1^)
A	142	4.9 × 10^7^	7.01	1.12 × 10^17^	8.24 × 10^−6^
B	120	8.2 × 10^7^	9.90	8.25 × 10^17^	3.33 × 10^−6^
C	122	1.0 × 10^8^	12.54	6.05 × 10^19^	4.66 × 10^−6^
D	111	3.7 × 10^7^	4.10	1.37 × 10^18^	8.21 × 10^−6^
E	139	1.7 × 10^7^	2.42	1.64 × 10^20^	1.98 × 10^−5^
F	112	3.1 × 10^6^	0.35	1.93 × 10^18^	4.32 × 10^−5^

**Table 5:** Comparison table the CuCrO_2_ data with other reported data.

Technique	Transmittance (%)	Resistivity (Ω cm)	Carrier Concentration (cm^−3^)	FOM (Ω^−1^)	Reference
**Sol–gel**	**71.4**	**2.42**	**1.64 × 10^20^**	1.98 × 10^−5^	**Sample E**
**Sol–gel**	**65.0**	**0.35**	**1.93 × 10^18^**	4.32 × 10^−5^	**Sample F**
RF-Sputtering	~63	~3	1.18× 10^21^	1.5 × 10^−7^	[41]
DC-MS	-	20	-	6.6 × 10^−7^	[13]
AA-MOCVD	52	-	-	1.4 × 10^−6^	[42]
RF-CS	~40	0.917	3.91 × 10^20^	2.07 × 10^−7^	[43]

DC-MS = DC magnetron sputtering, AA-MOCVD = Metal Organic CVD, RF-CS = RF-Cathodic Sputtering.

## Data Availability

Data available on request due to privacy/ethical restrictions.
